# Is asthma in the elderly different? Functional and clinical characteristics of asthma in individuals aged 65 years and older

**DOI:** 10.1186/s40733-019-0049-x

**Published:** 2019-03-19

**Authors:** Elena Curto, Astrid Crespo-Lessmann, María Victoria González-Gutiérrez, Santiago Bardagí, Concepción Cañete, Concha Pellicer, Teresa Bazús, María del Carmen Vennera, Carlos Martínez, Vicente Plaza

**Affiliations:** 1 Servicio de Neumología, Hospital de la Santa Creu i Sant Pau, Institut d’Investigació Biomédica Sant Pau, Carrer Mas Casanovas 90, 08041 Barcelona, Spain; 2 Servicio de Neumología, Consorci Sanitari del Maresme, Carrer de Cirera 230, 08304 Mataró, Barcelona, Spain; 3 Servicio de Neumología, Hospital General de l’Hospitalet, Av. Josep Molins 29, 08906 L’Hospitalet de Llobregat, Barcelona, Spain; 4 Servicio de Neumología, Hospital Comarcal Francesc De Borja, Avinguda de la Medicina 6, 46702 Gandia, València, Spain; 5 Servicio de Neumología, Hospital Universitario Central de Asturias, Av. Roma s/n, 3301 Oviedo, Spain; 6 Servició de Neumología, Hospital Clinic de Barcelona, Carrer de Villarroel 170, 08036 Barcelona, Spain; 7 Servicio de Neumología, Hospital Universitari Germans Trias i Pujol, Carretera de Canyet, s/n, 08916, Badalona, Barcelona, Spain

**Keywords:** Asthma, Elderly, Age, Phenotypes, Adults

## Abstract

**Background:**

The prevalence of chronic diseases in the elderly (> 65 years), including asthma, is growing, yet information available on asthma in this population is scarce.

Our objective is to determine the differential clinical and functional characteristics of the population > 65 years old with asthma included in the Integrated Research Programs of Asthma Databank of the Spanish Society of Pneumology and Thoracic Surgery (www.bancodatosasma.com).

**Methods:**

Retrospective comparative descriptive study of demographic, clinical and functional variables for 1713 patients with asthma categorized into 3 age groups as follows: adults aged < 65 years (A), younger elderly aged 65–74 years (B) and older elderly aged ≥75 years (C).

**Results:**

Predominant features of elderly patients with asthma (*N* = 471) were the female sex, fewer smokers, greater obesity, poorer lung function, and lower values of nitric oxide in exhaled air (*p* < 0.01). The most frequently associated comorbidity was gastroesophageal reflux. The highest doses of inhaled corticosteroids were by group A (60.8%). For the sample overall, 23.2% (*N* = 398) were being treated with omalizumab and 8.2% (*N* = 140) were corticosteroid-dependent (10.6% in group B). The highest percentage of patients receiving antileukotriene agents was in group B (42.9%).

**Conclusions:**

Asthma in adults aged> 65 is more severe and associated with greater comorbidity, which would indicate the need for a more integrated and multidimensional approach to asthma treatment for these patients.

## Backgrounds

Greater life expectancy has led to a remarkable increase in the prevalence of chronic diseases. According to the Spanish National Institute of Statistics (INE), the percentage of the population over 65 years of age, which currently stands at 18.2%, will be 24.9% in 2029 and 38.7% in 2064 [1]. Asthma has conventionally been considered a disease of children and young adults, but population ageing has led to a growing interest in the study of asthma in older people, as it is suspected that asthma prevalence may be underestimated [2], asthma-related morbidity and mortality may be greater [3] and socioeconomic and health costs are higher [4] in older population segments.

Little information is available in the specialized literature on the pathophysiological mechanisms that determine asthma-related airway inflammation in the elderly, but it is suspected that they may be different from the mechanisms at work in younger people [5]. Immunosenescence as described in the elderly [6] encompasses innate and adaptive changes in the immune response as a consequence of normal ageing. It can be assumed that this natural process combines with immune changes in the airways and comorbidities associated with advancing age to define the characteristics and particular phenotypes of elderly patients with asthma [7]. Some of these immunobiological characteristics are beginning to be known, but to date, almost exclusively from small studies [8, 9].

Older patients with asthma have a greater number of comorbidities, including obesity and gastroesophageal reflux (GERD) [10]. An added complication in the management of asthma is the concomitant use of treatments for comorbidities, along with possible technical difficulties in handling inhalers [11]. These patients are also likely to be underdiagnosed, due to factors such as a tendency to minimize symptoms [12], technical difficulties in performing spirometry diagnoses [13–15], and a lack of adapted reference values for functional test results [16].

This study was undertaken with the objective of determining possible differences in the clinical and functional characteristics of 3 groups -- older adults, younger elderly, and older elderly – with asthma.

## Methods

In this comparative retrospective study, data for all patients included from January 2008 to December 2013 in the asthma database of the Integrated Research Programme (PII) of the Spanish Society of Pneumology and Thoracic Surgery (SEPAR) (www.bancodatosasma.com) were extracted for descriptive analysis. Pulmonologists and allergists from seven Spanish centres (Hospital de la Santa Creu i Sant Pau, Hospital de Mataró, Hospital Francesc de Borja, HU Central de Asturias, Hospital Clínic, Hospital General de l'Hospitalet) contributed to this database with data on their outpatients, entered non-consecutively and when considered appropriate.

Analysed were demographic and clinical variables; disease control variables using the asthma control test (ACT) questionnaire [17]; number of hospital admissions in the previous year; oral corticosteroid treatment; therapeutic adherence (defined by the treating physician as good/fair/poor); functional variables (fraction of exhaled nitric oxide (F_e_NO following the 2005 recommendations of the American Thoracic Society/European Respiratory Society [41]) and forced expiratory volume in the first second (FEV_1_)); and the treatment received. Comorbidities were self-reported by the patients. 

Difficult-to-control asthma was defined as asthma that was insufficiently controlled despite an appropriate therapeutic strategy adapted to severity level [18]. Poorly controlled asthma was defined according to SEPAR guidelines [19] in force at the time of data collection and an ACT questionnaire score of < 20. The cut-off point of < 30 parts per billion (ppb) was used as reflecting normal F_e_NO values according to guidelines in force at the time of data collection [20]. Low, intermediate and high daily doses of inhaled corticosteroids -- beclomethasone propionate or equivalent -- were defined as < 500 μg, 500 μg-1000 μg and > 1000 μg, respectively. Adherence was defined as good, fair or poor according to the doctor’s criteria and the asthma education of the patient was similarly defined.

The study population of older adults was categorized into three population groups, as follows: < 65 years (group A, older adults), 65–74 years (younger elderly, group B), and ≥ 75 years (older elderly, group C) [21].

### Statistical analysis

Descriptive baseline values are reported as percentages and frequencies for the qualitative data and as means and standard deviations for the quantitative data. Categorical variables are described by contingency tables and using the chi-square test for the number and percentages of cases. Descriptive analysis was by age group and categorized by age. The level of statistical significance was set to 5% (α = 0.05) and SPSS (version 19.0) for Windows (SPSS, Inc., Chicago, Il, USA) was used for the statistical analysis.

## Results

A total of 1713 patients were analysed: 1242 (72.5%) aged < 65 years (group A), 282 (16.5%) aged 65–74 (group B), and 189 (11%) aged ≥75 years (group C).

### Sample characteristics

The overall sample (*N* = 1713) was predominated by women (68%; *N* = 1172) and mean body mass index (BMI) was 28.05 kg/cm^2^ (around a third (30%; *N* = 505) had a BMI greater than 30 kg/cm^2^). Of the sample, 14.3% (*N* = 245) had difficult-to-control asthma, and 71.7% (*N* = 1121) had allergic sensitization as demonstrated by the prick-test and/or specific immunoglobulin E (IgE). Regarding smoking habits, 63.8% of the sample (*N* = 1049) were non-smokers, 22.7% (*N* = 373) were ex-smokers, 8.6% (*N* = 142) were light smokers, 2.9% (*N* = 47) were passive smokers, and 1.9% (*N* = 32) were heavy smokers (more than 16 cigarettes/day according to the WHO (2003) definition).

Almost half (49.1%, *N* = 841/1615) of patients with asthma had rhinosinusitis and 16.5% (*N* = 208/1713) had nasal polyposis. Other comorbidities were GERD (66.5%; *N* = 123/185), followed by sleep apnea-hypopnea syndrome (OSA) (14.1%; *N* = 26/185), fibromyalgia (9.7%; *N* = 18/185), and inflammatory bowel disease (IBS) (1.1%; N = 2/185).

Current occupation (previous occupation for retired patients) was recorded for 48.6% of the sample (*N* = 833/1713), over half of whom (56%; *N* = 471/883) were/had been employed in high-risk occupations, mainly in the textile industry (*N* = 112/833), cleaning sector (*N* = 106/833), and agriculture (*N* = 31/833).

Nearly two thirds of the patients (63.1%; *N* = 1063/1684) had poor disease control according to the ACT questionnaire. Although most of the patients (87.2%; *N* = 1494/1713) had not required hospitalization in the previous year, 3.8% had previously been admitted more than once. Just over half (55%; *N* = 830/1495) of the patients had not received any systemic corticosteroid treatment for exacerbations, whereas 29.23% of patients had received this treatment more than twice in the previous year.

Regarding maintenance treatment, the most common inhaled corticosteroid at baseline was budesonide (54%; *N* = 935/1713), usually at intermediate doses (49%), and the most typical long-acting beta adrenergic agonist (LABA) was formoterol (46.9%; *N* = 803/1713). As adjuvant therapies, 33.9% of the sample were being treating with antileukotriene agents, 4.5% with theophylline, 23.2% with omalizumab, 0.6% with immunotherapy, and 8.2% with fixed-dose oral corticosteroids.

Treatment adherence was rated as good (70.9%), poor (13.8%) or fair (5.2%) by attending physicians at the time of data collection, and 60.2% (*N* = 889/1476) were considered to have received some type of asthma education. As for lung function, 74.5% had FEV_1_ > 65 and 9.2% had FEV_1_ < 50%.

### Characteristics by age group

Table 1 summarizes the clinical and functional characteristics (with statistical significance values) for the 3 age groups: older adults (group A), younger elderly (group B) and older elderly (group C). Women predominated in the 3 groups in similar proportions (although not to a statistically significant degree), obesity was higher in the 2 elderly groups (B and C), there were more smokers/ex-smokers in the older adult group (A), and lung function was more impaired, whereas F_e_NO values were lower, in the 2 elderly groups (B and C).

**Table 1:** Characteristics of patients with asthma by age group

Variable	Group A: < 65 years *N* = 1240 (72.5%)	Group B: 65–74 years *N* = 282 (16.5%)	Group C: ≥75 years *N* = 189 (11%)	p
Sex (% women)^a^	67.8%	68.8%	72.9%	0.397
Active smokers^a^	13.7% (*N* = 163/1185)	3.7% (N = 10/272)	0.5% (N = 1/186)	<0.001
Ex-smokers^a^	23.8% (N = 282/1185)	21.3% (*N* = 58/272)	17.7% (*N* = 33/186)	<0.001
Obese^a^ (BMI > 30 kg/cm^2^)	25.5% (*N* = 299/1171)	45.8% (*N* = 125/273)	44.3% (*N* = 82/185)	<0.001
Diagnosed with difficult-to-control asthma^a^	15.3% (*N* = 190/1240)	14.2% (*N* = 40/282)	7.9% (*N* = 15/189)	0.027
FEV_1_ < 50%^a^	7.0% (*N* = 74/1064)	13.7% (*N* = 34/249)	16.7% (N = 28/163)	<0.001
FEV_1_ < 50%	54.4% (N = 74/136)	25% (N = 34/136)	20.6% (N = 28/136)	<0.001
F_e_NO > 30 ppb	89.9% (*N* = 160/178)	8.4% (N = 15/178)	1.7% (N = 3/178)	<0.001
Received ≥2 corticosteroid cycles in the previous year	70.9% (*N* = 310/437)	19.7% (*N* = 86/437)	9.4% (*N* = 41/437)	0.106
Treated with high doses of inhaled corticosteroids^b^	60.8% (*N* = 236/388)	20.9% (*N* = 81/388)	18.3% (*N* = 71/388)	0.045
Treated with antileukotriene agents	33% (*N* = 387/1173)	42.9% (*N* = 115/268)	27.8% (*N* = 52/187)	0.001
Treated with omalizumab	76.4% (*N* = 304/398)	18.1% (*N* = 72/398)	5.5% (*N* = 22/398)	0.288

Abbreviations: *BMI* body mass index, *F*_*e*_*NO* fraction of exhaled of nitric oxide, *FEV*_*1*_ forced expiratory volume in the first second

^a^indicates analysed categorized by age group

^b^> 1000 μg/day beclomethasone dipropionate or equivalent

Older adults (group A) received more cycles of oral corticosteroids (70.9% ≥2 cycles in the previous year) and higher doses of inhaled corticosteroids andwere mainly treated with omalizumab (76.8% *N* = 337).

The diseases most frequently associated with the age groups (adjusted for age) were GERD, predominating in group C (72.2%), OSA, predominating in group B (19.4%), and fibromyalgia, predominating in group A (11%).

## Discussion

Our study demonstrates that asthma is clinically different in elderly patients. The fact that asthma severity and comorbidities are higher than in other adults needs to be taken into account to ensure multidimensional management when treating these patients.

In the past, asthma was considered predominantly a disease of childhood, but recent epidemiological studies indicate that it can affect people at any age, and is, in fact, very prevalent in the adult population (4.5–12.7%) [22]. According to current demographic trends, a 100% increase in the number of people aged over 65 is expected in the coming decades [22, 23]. It would therefore be reasonable to assume that there will be an increase in the impact of asthma on older population segments in the near future.

With respect to the general characteristics of the studied population (*N* = 1713), 68% were women; 27.5% were aged over 65 years; 63.8% were non-smokers; 49% had rhinosinusitis and 16.5% had nasal polyposis; 10.8% had comorbidities; 74.5% had FEV_1_ > 65%; 70.9% were good adherents to treatment; and 14.3% had difficult-to-control asthma and 63.1% showed poor asthma control (ACT< 20). The high percentage of poor control is probably due to the fact that many patients were included on their first visit to the specialist, with poor control often being the very reason for referral. Furthermore, the high percentage of difficult-to-control asthma may be explained by the fact that many of the doctors who input the data were asthma specialists to whom severe cases were referred.

While better tools are available nowadays to measure therapeutic adherence by patients using inhalers, such as the test of adherence to inhalers (TAI) [24], electronic monitoring of prescribed medication collection and the use of electronics devices, at the time of creating the database these tools were not available. Adherence data was therefore compiled, as good, fair or poor, according to the doctor’s criteria. While adherence for our sample was rated as good in 70.9% of the cases, this value is probably overestimated because the method used to assess adherence was the subjective opinion of the doctor.

Obesity, whose prevalence is increasing worldwide, represents a risk to the health of people at all ages, and not only because obese people are at an increased risk of asthma [25], but because they have more asthma symptoms, more frequent and serious exacerbations [26], poorer control [27], poorer response to various asthma medications [28], and a reduced quality of life [29]. However, because obesity causes significant changes in normal lung physiology (reduced expiratory reserve volume and, therefore, functional residual capacity), it has been demonstrated that obesity can be a confounder for a false diagnosis of asthma -- a fact which needs to be borne in mind in the diagnosis of asthma in obese individuals. Aaron et al. [30], for instance, in a prospective study of 540 patients with asthma diagnosed by a physician, subsequently assessed by spirometry with a bronchodilator test, methacholine challenge test and asthma treatment withdrawal, concluded that nearly a third (31.8%) of obese people were wrongly diagnosed as having asthma. We found that the elderly patients with asthma in our sample were more prone to obesity, which, as a risk factor and an indicator of poor asthma control, would indicate the need for obesity to be taken into account in their therapeutic management. Also, it is important to consider that an important cumulative dose of corticosteroids will lead to weight gain, and higher risk of OSA, wich is even more important in elder patients with a longer asthma trajectory. In these patients, the symptoms of asthma combined with those of other comorbidities (like arthrosis, or congestive hearth failure) increase the risk of a sedentary lifestyle and therefore, overweight.  Data on cardiovascular diseases and depression, which probably also play a role, were not collected in our study. The collected data from comorbidities of upper airway like rhinitis and nasal polyps are similar in general from those reported by Tiotiu et al [42] and other series. We did not collect data of vocal cord dysfunction, wich could also yield interesting results.

With respect to lung function, our study found that the elderly patients (> 65 years) had a poor pulmonary function, measured as FEV_1_. This result is of interest because, as is known, forced vital capacity (FVC) and FEV_1_ decrease with age from a maximum reached between 20 and 25 years [31]. This physiological decrease, combined with a poor pulmonary function, may alter symptoms and quality of life in elderly patients. Since forced vital capacity (FVC) data were not collected, the FEV_1_/FVC ratio could not be calculated. Given the high percentage of obesity observed in the elderly in our sample, this ratio would have allowed us to assess the presence or absence of obstruction so as to rule out mixed ventilatory impairment [32, 33].

With regard to the low F_e_NO values in the elderly in our sample, this may be explained by the fact that the group of adults aged < 65 years had a greater allergenic component, corroborated by the higher percentage being treated with omalizumab. Although some studies have found no differences in F_e_NO values between patients with and without obesity [34], others have reported that obese people have higher F_e_NO values to a statistically significant degree [35]. However, the fact that studies to date conducted in elderly populations have been based on small samples possibly explains the contradictory results [36–40]. In our study, patients > 65 years old, despite having relatively higher obesity rates, had lower F_e_NO values. This finding corroborates a study of 30 patients aged ≥65 years with stable asthma followed up for a year with evaluations every 3 months, which found that F_e_NO values were not elevated and did not correlate with demographics, comorbidities, treatment, symptoms or spirometry values. In fact, the suggestion by the authors of that study, that routine measurements of F_e_NO values may not be clinically useful for elderly asthmatics [36], is corroborated by our results. Likewise, the fact that the elderly patients had a lower allergenic component may also have a bearing on their lower F_e_NO values. However, while age itself may be a factor that could affect the cut-off point for a normal F_e_NO value, perhaps its usefulness in the diagnosis and follow-up of these patients needs to be reassessed.

Another aspect of interest is the economic burden of asthma in the elderly population. A study conducted by Plaza et al. [4] showed that the direct costs of managing asthma in the elderly were double those for non-elderly adults (an annual average of USD 1490 vs USD 773), mainly due to higher hospitalization and drug costs. Although our study did not assess asthma costs, the higher economic burden can be inferred from our results regarding greater disease severity and morbidity in the elderly in our sample.

The main strengths of this study are that the sample was large (1713 patients), the data were collected by asthma specialists, and many variables were analysed. As limitations, many variables were not completed by the professionals (because they did not have particular data or tests at their centre or due to a lack of time), the analysis was only transversal and descriptive, and the study may be biased by the fact that the sample may have had more severe asthma, given that the doctors who input the data were asthma specialists who more frequently treat more severe cases. Another fact not reflected in our data is that a diagnosis of asthma is generally recognized to be ruled out in a significant percentage of patients referred to an asthma unit. Finally, our analysis was based on a relatively old database (2008–2013) and therefore lacks certain information of current interest, such as objectively measured adherence to treatment (e.g., TAI, electronic prescription monitoring), and also excluded certain data that was not collected (such as years with asthma, phenotype classification, smoking-related factors, etc).

## Conclusion

Our main result is that the functional and clinical characteristics of asthma in elderly patients (> 65 years) and older adults (< 65 years) are different, and that this difference should be borne in mind, along with age-related factors, in routine practice in order to achieve optimal disease control. Given that the fastest growing age band is the population aged > 65 years, prospective studies are necessary to assess asthma behaviour and impact in the elderly.
